# Potential role for vitamin D vs. intermittent fasting in controlling aquaporin-1 and aquaporin-3 expression in HFD-induced urinary bladder alterations in rats

**DOI:** 10.3389/fmolb.2023.1306523

**Published:** 2024-01-22

**Authors:** Hend M. Hassan, Randa El-Gamal, Walaa. H. E. Hamed, Ola Ali Habotta, Mervat Samy, Rasha Elmowafy, Eman Mohamed ElNashar, Mansour Abdullah Alghamdi, Rashid A. Aldahhan, Khulood Mohammed Al-Khater, Mohammed A. Alshehri, Magda E. Ahmed

**Affiliations:** ^1^ Department of Human Anatomy and Embryology, Faculty of Medicine, Mansoura University, Mansoura, Egypt; ^2^ Department of Human Anatomy and Embryology, Faculty of Medicine, New Mansoura University, Mansoura, Egypt; ^3^ Department of Medical Biochemistry and Molecular Biology, Faculty of Medicine, Mansoura University, Mansoura, Egypt; ^4^ Medical Experimental Research Centre (MERC), Faculty of Medicine, Mansoura University, Mansoura, Egypt; ^5^ Department of Medical Histology and Cell Biology, Faculty of Medicine, Mansoura University and New Mansoura University, Mansoura, Egypt; ^6^ Forensic Medicine and Toxicology Department, Faculty of Veterinary Medicine, Mansoura University, Mansoura, Egypt; ^7^ Department of Clinical Pharmacology, Faculty of Medicine, Mansoura University, Mansoura, Egypt; ^8^ Department of Anatomy, College of Medicine, King Khalid University, Abha, Saudi Arabia; ^9^ Genomics and Personalized Medicine Unit, College of Medicine, King Khalid University, Abha, Saudi Arabia; ^10^ Department of Anatomy, College of Medicine, Imam Abdulrahman Bin Faisal University, Dammam, Saudi Arabia; ^11^ Nephrology Section, Internal Medicine Department, College of Medicine, King Khalid University, Abha, Saudi Arabia

**Keywords:** high-fat diet, urinary bladder, AQP-1, AQP-3, intermittent fasting and vitamin D

## Abstract

**Background:** High-fat diet-induced obesity is linked to suppression of aquaporins (AQPs) expression in different tissues. Both vitamin D and intermittent fasting were identified to enhance AQPs expression. In the urinary bladder, AQP-1 and AQP-3 mRNA transcripts were identified. Vitamin D has an impact on a variety of genes that encode proteins that control cell proliferation, differentiation, and death.

**Aim:** To assess potential benefits of vitamin D and intermittent fasting (IF) and to explore alterations to the urinary bladder triggered by high-fat diet (HFD) in a rat model of obesity.

**Methods:** Each of the 4 groups contained six adult male albino rats; control: a standard rodent chew for 12 weeks, HFD: HFD and fructose were administered orally via gastric gavage for 12 weeks, and vitamin D: HFD and fructose were administered orally for 8 weeks, then 4 weeks of intraperitoneal injection of vitamin D (5 microns/Kg/2 days) and IF group: Received intraperitoneal injections of vitamin D (5 microns/Kg/2 days) for 4 weeks after consumption of HFD and fructose orally for 8 weeks. The serum lipid profile was conducted at end of the experiment. In the bladder homogenates, the levels of oxidative stress indicators were assessed. Quantitative real-time PCR was performed on recently collected bladder samples. AQP-1 and AQP-3 immunohistochemistry was done.

**Results:** When compared to the HFD group, the vitamin D and IF groups both demonstrated a substantial improvement in histopathological, immunohistochemical, biochemical, and molecular markers.

**Conclusion:** In all examined parameters, IF exceeded vitamin D as a preventive factor for the urinary bladder deterioration.

## Graphical Abstract

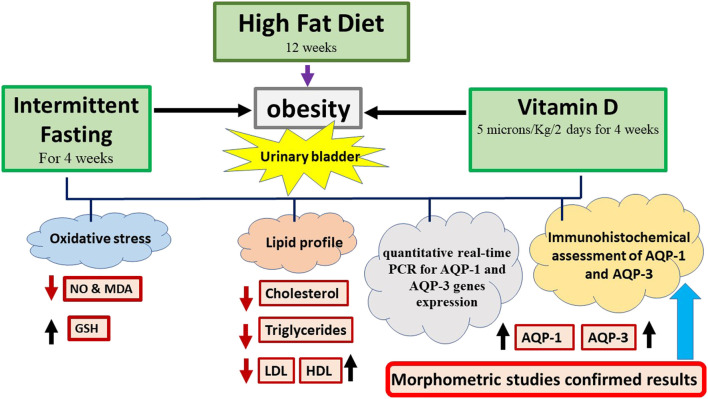

## 1 Introduction

Globally, obesity has emerged as a serious public health concern. The health of several countries is threatened by the rising incidence of body mass index (BMI) and the mortality that follows. (Okati-Aliabad et al., 2022). Obesity can be complicated by diabetes, stroke, pulmonary dysfunction, sleep apnea, coronary heart disease, and more (Kinlen et al., 2018). Obesity is regarded as a low-grade inflammatory disease because the majority of obese patients have higher levels of inflammatory markers such tumour necrosis factor alpha and interleukin-6 (Mraz and Haluzik, 2014).

Rats fed a high-fat diet (HFD) accumulate more fat, have higher blood sugar levels, and have higher blood triglycerides; as a result, HFD-fed rats regularly serve as models to investigate obesity (Marques et al., 2016). Obesity introduced by HFD is linked to a higher risk of metabolic illnesses such as insulin resistance, hypertension, dyslipidaemia, and others. (Polsky and Ellis, 2015). Additionally, it increases the harm to various organs of the urogenital system (Aizawa et al., 2013; Furriel et al., 2014).

Obesity has been associated with lower urinary tract, according to studies conducted in both experimental animals and humans (Oberbach et al., 2013). Many unpleasant urological symptoms associated with obesity indicate functional bladder involvement (Daneshgari et al., 2017). Studies on the physical effects of HFD on detrusor muscle fibres have shown that their actions are disrupted (Lambertucci et al., 2012).

Aquaporins (AQPs) are proteins that allow osmotic pressures to transfer water across cells. On exposure to an osmotic gradient without AQPs, lipid bilayers’ diffusional permeability of the lipid bilayers decreases (An and Zhu, 2021). According to permeability, AQPs are classified into permeable to water, glycerol, urea, and/or tiny solutes (Ishibashi et al., 2014). Eight of the 13 AQP isoforms have been identified in human tissues (Seyahian et al., 2020). They are present in numerous tissues that retain fluid and have a role in transepithelial fluid transport, urine concentration, and fluid secretion (Sales et al., 2013).

Aquaporin-1 (AQP-1) is expressed in the descending limb of the kidney, the proximal tubules, and the red blood corpuscles (Gannon and Carati, 2003). It is located in the urinary bladder’s uroepithelium, capillaries, arterioles, and venules of the urinary bladder (Kim et al., 2010).

In addition, aquaporin-3 (AQP-3) is located along the epithelial lining of the urinary tract. It is only found in the plasma membrane of the basal and intermediate cells (Lionarons et al., 2019). AQP-1 and AQP-3 mRNA transcripts were detected, in the juvenile porcine bladder, and confirmed their protein expression with immunohistochemistry (Vahabi et al., 2015).

In the juvenile porcine bladder, AQP-1 and AQP-3 mRNA transcripts were found, and immunohistochemistry validated their protein expression (Vahabi et al., 2015).

Due to their involvement in tissue lipid accumulation and oxidative stress, two of the most important components of insulin resistance (IR), AQPs become important participants in obesity-induced IR (Galli et al., 2021). For the treatment of obesity and the control of fat deposition, it may be possible to regulate the AQP expression (Da Silva and Soveral, 2017).

A fat-soluble vitamin, vitamin D can be obtained from a variety of dietary sources. In addition, UV rays that pierce the skin and facilitate its activation cause it to be created inside the body (Ross et al., 2011). By controlling the synthesis of inflammatory cytokines and immune cells, which are essential for the pathophysiology of many immune-related disorders, vitamin D plays a significant role in modulating the inflammatory system (Ross et al., 2020). According to Larsson and Voss (2018), vitamin D may defend against the harm caused by obesity to enteric neurons.

Additionally, according to Fu et al. (2017), vitamin D controls the expression of AQP in the kidneys of mice. Vitamin D exerted its protective impact by reducing inflammation and fibrosis in a rat model of acute renal damage driven on by gentamicin. This clearly raised AQP-1 (Park et al., 2010). It was also reported that vitamin D strengthens the epithelium of the urinary bladder (Mohanty et al., 2020).

It is interesting to note that a strong link exists between low vitamin D levels and obesity. It takes higher doses of vitamin D than are frequently recommended for the general population to treat low vitamin D concentrations in obese people. According to Vanlint (2013), calcium and vitamin D are thought to regulate adipocyte death (apoptosis), adipogenesis, and lipid metabolism.

The general public is very interested in intermittent fasting (IF) as an alternative to the conventional daily energy restriction strategy for treating obesity and related diseases. IF is the practice of constantly going for extended periods of time (16 h) without eating or consuming very little energy, followed by regular meals of normal dietary intake (Mattson et al., 2017). In those with obesity and type 2 diabetes, it is linked to weight loss, the modification of abdominal circumference, and an improvement in the control of glycaemic levels. It also has positive effects on the lipid profile. (Morales-Suarez-Varela et al., 2021). Furthermore, IF modifies oxidative stress by decreasing mitochondrial ROS generation and increasing endogenous antioxidant activity (Lanza et al., 2012).

Furthermore, IF was shown to be helpful in treating certain conditions through the regulation of AQPs expression in different tissues, e.g., brain, peritoneum, salivary glands and liver (Zhang et al., 2017; Taha et al., 2022).

The best that we can tell, most recent studies concentrated on the impact of obesity on the physiological functions of the urinary bladder without mentioning the morphological changes underlying these deteriorations. The bladder has not been sufficiently examined when exposed to HFD. Additionally, no prior studies compared the beneficial effects of IF *versus* vitamin D on the HFD-induced model of obesity.

In order to investigate these changes, the current study used a rat model of obesity produced by HFD. Moreover, the advantages of vitamin D supplementation *versus* IF on the urinary bladder were investigated.

## 2 Materials and methods

### 2.1 Sample size calculation

G*Power software (version 3.1.9.7) was used to determine the sample size. A total of 24 albino rats, six per group from each of the four groups whose means were to be compared, were used in the one-way ANOVA test. The F test with a significance level of 0.05 gave the 24-rat sample a power of 80% to identify mean differences compared to the alternative of equal means. The effect size η^2^ = σm^2^/(σm^2^ + σ^2^) 0.40, represents the extent of the variation in the means.

### 2.2 Animals used

Twenty-four adult male albino rats weighing 200–220 g and aged 12–14 weeks were utilised in the study. The experiment was conducted at Mansoura Faculty of Veterinary Medicine where the animals were obtained. Rats were maintained at a constant temperature of 20°C, a humidity level of 50%, and a dark/light cycle of 12 h/12 h. They were free to get water and a typical diet.

### 2.3 Ethical approval

The work was approved by the Medical Research Ethical Committee, Faculty of Veterinary Medicine, Mansoura (code number: R/134). All feasible steps were taken to reduce both the number of animals utilised and their suffering.

### 2.4 Chemicals used

Vitamin D was obtained from El-Gomhorya Company for Medicines, Mansoura, Egypt. From abcam (Egypt), antibodies against AQP-1 (AB2219, 1: 1000 dilution) and AQP-3 (A0303, 1: 200 dilution) were purchased. Standard rodent chow and high fat diet were obtained from CLEA Japan.

### 2.5 Study plan

Following acclimatisation for 2 weeks, the rats were enrolled randomly into 4 groups: **Control** in which rats fed a standard rodent chew for 12 weeks containing 51.93 g of carbohydrates, 3.03 g of fat, 20.50 g of protein, 4.17 g of crude fibre per 100 g of diet, and 3.00 kcal per gramme of energy (Ahmed et al., 2017). Rats in the HFD group received a 12-week HFD diet (60 percent fat, 20 percent carbohydrates, and 20 g of fructose dissolved in 100 mL of tap water). Vitamin D group in which rats received intraperitoneal injections of vitamin D at a dose of 5 microns/kg/2 days for 4 weeks after being fed HFD and fructose for 8 weeks (Yin et al., 2012a). IF group in which rats were given an HFD and fructose diet for 8 weeks, after which 4 weeks of intermittent fasting (24 h of feeding followed by 24 h of fasting) were implemented (Zhang et al., 2017).

### 2.6 Sacrifice and blood sampling

The rats fasted for 12 h during the 12th week, anaesthetised using chloral hydrate (300 mg/kg, intraperitoneal) and then blood was drawn from the tail vein in EDTA-free tubes. Blood was allowed to clot at room temperature and serum was separated using a centrifuge for 15 min at 3000 rpm (Hettich universal 32A, Germany). Subsequently, serum samples were stored at −20°C until analysis was complete. The bladder was carefully excised, washed with cold normal saline (0.9% NaCl solution), and then dried on filter papers. The bladder was divided into three parts. A part was embedded in formalin to prepare paraffin sections (4 µm thick) to be stained with haematoxylin and eosin (H &E**)** and AQP-1 and AQP-3 immune stains. The second part was kept fresh to prepare homogenates to be used for biochemical studies. The last part was kept in RNA later for quantitative real-time PCR.

### 2.7 Assessments

#### 2.7.1 Assessment of the serum lipid profile

The following commercially available kits were used to measure serum levels of total cholesterol (TC), triglycerides (TG), low-density lipoproteins (LDL) and high-density lipoproteins (HDL): total cholesterol assay endpoint kit (MG, cat. no. MG230001), triglyceride assay endpoint kit (MG, cat. no. MG314001) and HDL cholesterol assay endpoint kit (MG, cat (Burtis, 1999; Shih et al., 2000; Young and Friedman, 2001). In addition, the formula LDL cholesterol = total cholesterol - HDL cholesterol - (triglycerides/5) was used to calculate LDL cholesterol (Friedewald et al., 1972). The Erba CHEM-7 apparatus and the manufacturer’s instructions (ERBA Diagnostics, India) were used for all colorimetric assays.

#### 2.7.2 Evaluation of nitric oxide (NO), malondialdehyde (MDA) and reduced glutathione (GSH) in the urinary bladder tissue homogenates

Using a tissue homogenizer (Heidolph Silent Crusher M, Germany), a sample of each rat’s urinary bladder was homogenized in 10 mL of cold buffer (50 mM potassium phosphate, pH 7.5) per gram of tissue. After that, the homogenate was centrifuged at 4°C for 15 min at 4,000 rpm. According to the instructions provided by the manufacturer (Bio-Diagnostics, Egypt), the quantities of nitric oxide (NO), malondialdehyde (MDA), and reduced glutathione (GSH) in the homogenate supernatant were measured using a colorimetric method (Pires et al., 2014; Barros et al., 2017; Bhidwaria and Ashwlayan, 2017).

#### 2.7.3 mRNA quantification by real-time reverse transcription-PCR (qRT-PCR)

RNA Later (10 ul per 1 mg tissue sample) (Qiagen, Germany) was used to store urinary bladder tissue samples. These samples were first stored overnight at 2°– 8°C then stored at −80°C until processing. After processing, the tissue samples were homogenised using five strokes of liquid nitrogen. Complete cellular RNA was isolated using the QIAzol reagent (Qiagen, Germany). Thermo Scientific NanoDrop One (United States) evaluated the concentration and purity of the RNA yield. First-strand cDNA was produced from 1ug of RNA (Applied Biosystems, United States) using the Revertaid First Strand cDNA Synthesis Kit (Thermoscientific, United States) and a Proflex Thermal Cycler.

The temperature was adjusted to 25°C for 5 min for primer annealing, 42°C for 60 min of reverse transcription, and 70°C for 5 min for inactivation. The cDNA templates were amplified using real-time PCR equipment (Azure Cielo 6, United States). A total of 20 μL were used in the reaction: 10 μL of HERA SYBR green PCR Master Mix (Bioline, UK), 1 μL of cDNA template, 2 μL of gene primer (10 pmol/L), and 7 μL of nuclease-free water. The thermal profile was then put through 40 cycles of annealing and extension at 60°C for 30 s each after being denaturized at 95°C for 2 minutes.

As a reference gene, glyceraldehyde-3-phosphate dehydrogenase (GAPDH) was used. The primer pair sequences that were utilised are shown in (Table 1). The specificity of the primer was evaluated using the Primer-BLAST programme (https://www.ncbi.nlm.nih.gov/tools/primer-blast/). The company Vivantis (Vivantis Technologies, Malaysia) provided some primer sets. Melting curve analysis was used to confirm the results of the PCR were specific. In order to quantify the fold change in gene expression using the 2^−ΔΔCT^ approach, relative gene expression levels were expressed as ΔCt = Ct target gene - Ct housekeeping gene (Livak and Schmittgen, 2001).

**TABLE 1:** The sequence of rat primers used in qRT-PCR analysis.

Gene	Sequence	Product size	RefSeq	References
Aquaporin 1 (AQP-1)	Forward: GCT​GTC​ATG​TAT​ATC​ATC​GCC​CAG	107 bp	NM_012778.2	Bouley et al. (2009)
	Reverse: AGG​TCA​TTT​CGG​CCA​AGT​GAG​T			
Aquaporin 3 (AQP-3)	Forward: AAG​CTG​CCC​ATC​TAC​ACA​CT	234 bp	NM_031703.2	https://www.ncbi.nlm.nih.gov/tools/primer-blast/
	Reverse: GGC​CAG​CAC​ACA​CAC​AAT​AA			
Glyceraldehyde-3 phosphate dehydrogenase (GAPDH)	Forward: TGG​GAA​GCT​GGT​CAT​CAA​C	78 bp	NM_017008.4	Tripathi et al. (2014)
	Reverse: GCA​TCA​CCC​CAT​TTG​ATG​TT			

### 2.8 Histopathological examination of urinary bladder sections

For routine histopathological examination (Mahar et al., 2021). In the present study, we evaluated the histopathological changes in all studied groups. A semiquantitative score was given based on the absence (=0) or presence (=1) of each of the histopathological finding (vacuolated cells, dilated vessels and inflammatory cellular infiltrate, epithelial ulceration, and widely separated muscle bundles) and we gave them a total score for each section.

#### 2.8.1 Immunohistochemical detection of AQP-1 and AQP-3 in the urinary bladder

Paraffin sections (4 μm thick) were deparaffinized in xylene and rehydrated with 0.03% H2O2 to block the endogenous peroxidases. After antigen retrieval in a microwave for 20 min with a pH-neutral sodium citrate buffer, the antigen was blocked with 5% bovine serum albumin in tris buffered saline. The sections were then treated with a primary antibody against AQP-1 (AB2219, 1: 1000 dilution) and AQP-3 (A0303, 1: 200 dilution) for an additional overnight period at 4°C. The ABC kit was used to detect the reaction according to the manufacturer’s instructions (Sigma-Aldrich, St. Louis, MO, United States). The sections were then dried, mounted with a synthetic glue medium, and counterstained with haematoxylin, then analysis of tissue sections was analyzed by light microscopy (Hassan et al., 2022).

#### 2.8.2 Measurement of the area percentage of AQP-1 and AQP-3 positive reactions in the urinary bladder

Images were taken using a digital camera (Toucan type BX53, Japan) linked to a computer and a light microscope (Olympus model BX53, Tokyo, Japan). For each rat in each group, the urinary bladder was examined in five randomly placed, 4 µm thick slices using a 40x lens and a ×400 magnification (area: 0.071mm2). The area fraction of AQP1 and AQP3 immune expression was the estimated parameter. ImageJ (Fiji) was used for computerized image analysis. Brownish coloration was indicative of immune expression. Three separate-colored images were created using a color deconvolution plug-in with the H-DAB vector as the chosen color: green, brown, and blue. The DAB images, which were colored brown, were calibrated by determining the area fraction (Hassan et al., 2022).

### 2.9 Statistical analysis

The data were analysed using IBM SPSS for Windows 10 (Chicago, IL, United States), version 26.0. Using the Shapiro-test Wilk’s, normalcy was examined. Data that was regularly distributed was described using the mean and SD. At the (0.05) level, the significance of the obtained results was evaluated. One-way analysis of variance (ANOVA) was used to compare quantitative data among the four research groups, and the *post hoc* Games-Howell test was used to evaluate two groups against one another. Games-Howell *post hoc* test is effectively a Welch’s version of Tukey’s test and it works well with small sample sizes.

## 3Results

### 3.1 Vitamin D *versus* intermittent fasting effects on lipid profile

**FIGURE 1:**
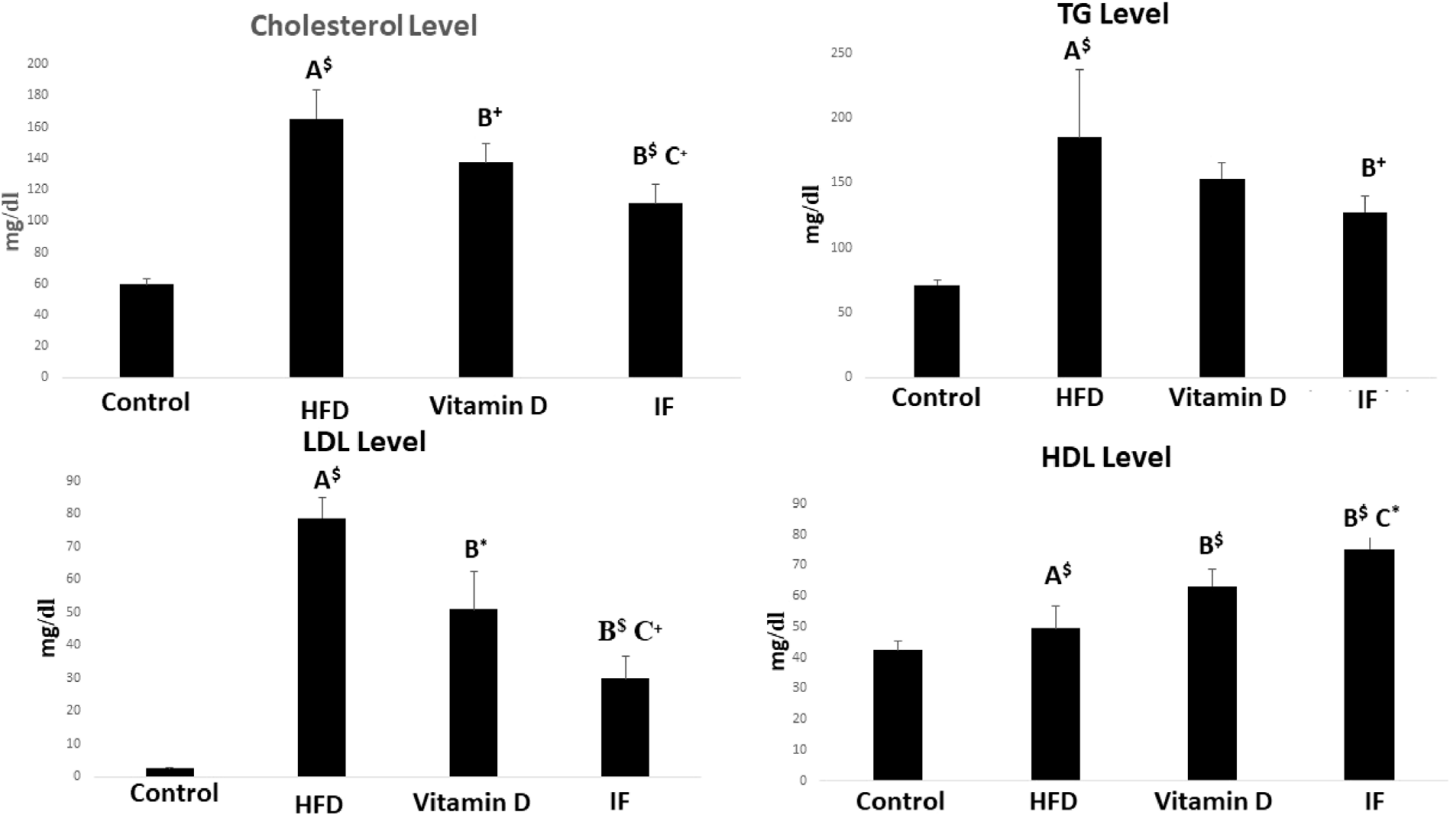
Vitamin D versus intermittent fasting effects on the lipid profiles. The Games Howell post-hoc test was applied after a one-way ANOVA. A: Relevance for the control. B: Relevance for HFD. C: Significance for the vitamin D group. +: *p* < 0.05, *: *p* ≤ 0.01, $: *p* ≤ 0.001.

The HFD group’s serum levels of TC, TG, and LDL-C were all noticeably greater than those of the control group (*p* = 0.001 for each). Vitamin D group revealed that HDL-C increased significantly (*p* = 0.01), TC and LDL-C decreased dramatically (*p* = 0.04, *p* = 0.001 respectively) when compared to HFD group. Additionally, when compared to the HFD group, IF markedly reduced TC (*p* = 0.001), TG (*p* = 0.04), and LDL-C (*p* = 0.001) while sharply raising HDL-C (*p* = 0.001). Additionally, vitamin D group TC, LDL-C, and HDL-C levels were considerably higher than those of the IF group (*p* = 0.05, 0.007, 0.03 respectively) (Figure 1).

### 3.2 Vitamin D *versus* intermittent fasting effects on oxidative stress indicators

**FIGURE 2:**
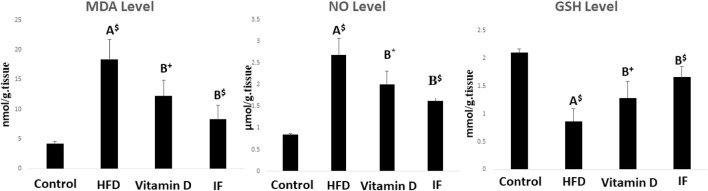
Indicators of oxidative stress in rats fed the HFD and effects of vitamin D versus intermittent fasting. The Games Howell post-hoc test was used after a one-way ANOVA. A: Relevance for control. B: Relevance for HFD. +: *p* < 0.05, *: *p* ≤ 0.01, $: *p* ≤ 0.001.

After 12 weeks of feeding on HFD, the rats’ MDA and NO levels statistically increased in comparison to the control rats, but their GSH levels statistically decreased (*p* = 0.001 for all). However, when compared to the HFD group, vitamin D caused considerably lower levels of MDA (*p* = 0.02), NO (*p* = 0.01), and greater levels of GSH (*p* = 0.05). Additionally, when compared to HFD group, IF had a significantly lower level of MDA and NO and a significantly higher level of GSH (*p* = 0.001 for all) (Figure 2).

### 3.3 Vitamin D *versus* intermittent fasting effects on AQP-1 and AQP-3 gene expression in bladder tissues

**FIGURE 3:**
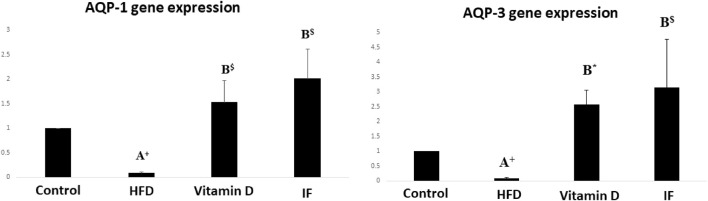
Gene expression histograms for AQP-1 and AQP-3, displaying the means and standard deviations of the fold change. Test procedures: one-way ANOVA, then a Games Howell post hoc analysis. A: Relevance for control. B: Relevance for HFD. +: *p* < 0.05, *: *p* ≤ 0.01, $: *p* ≤ 0.001.

HFD group demonstrated significantly lower expression of the AQP-1 and AQP-3 genes as compared to the control group (*p* = 0.02 for both). When vitamin D was administered, AQP-1 and AQP-3 gene expression significantly increased (*p* = 0.001 and *p* = 0.006, respectively). Additionally, IF dramatically raised the expression of AQP-1 and AQP-3 in comparison to HFD group (*p* = 0.001 for both) (Figure 3).

### 3.4 Histopathological results of urinary bladder tissue sections in study groups

**FIGURE 4:**
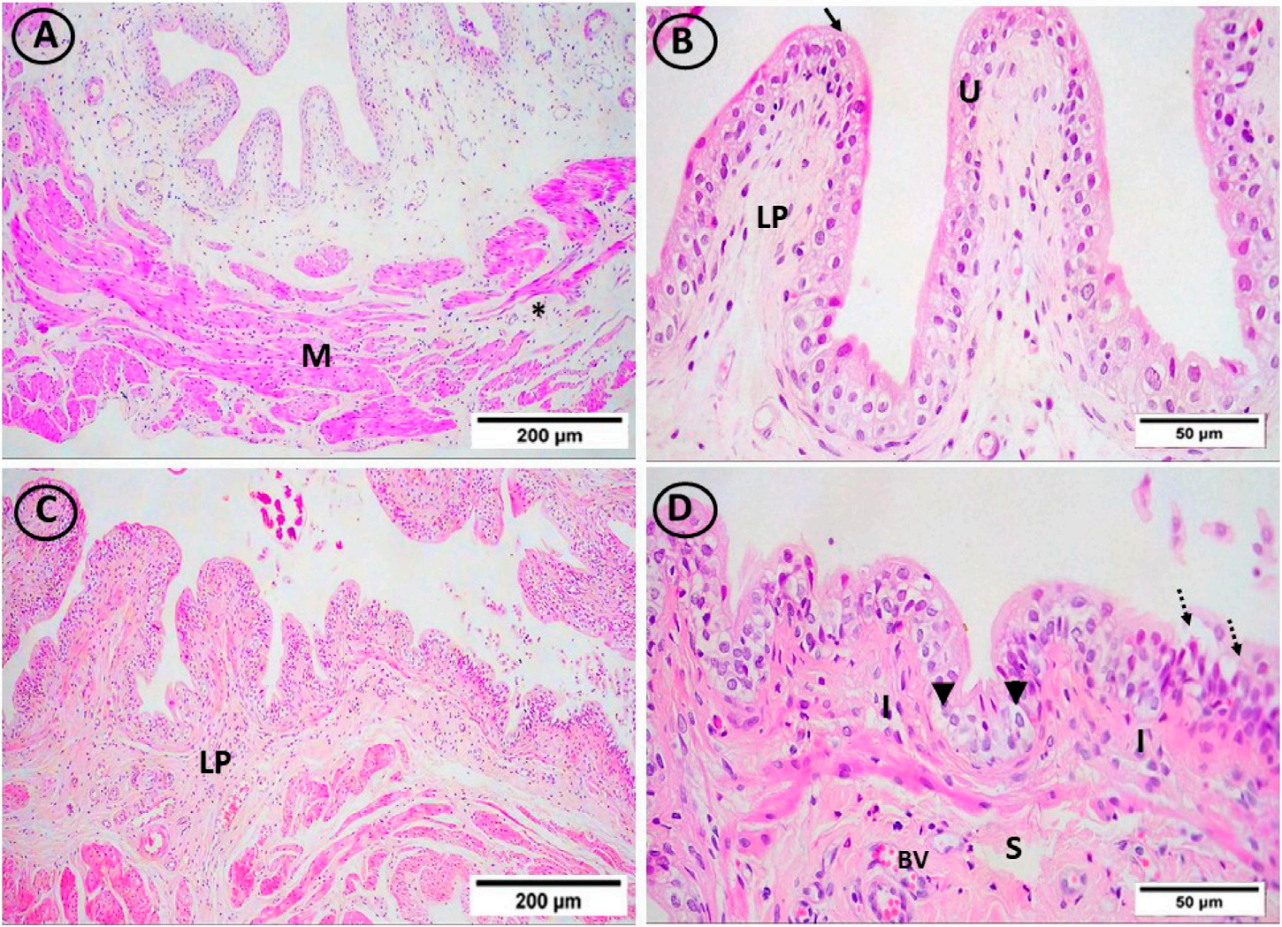
H & E-stained bladder sections. **(A, B)** The mucosa in the control group had a highly folded appearance (arrows), was lined with transitional urothelium (U), and was underlain by lamina propria (LP) and smooth muscle bundles (M), which were separated by connective tissue bundles (star) and ran in opposing directions. **(C, D)** The transitional epithelium thickness appeared to have reduced, and the HFD group showed isolated areas of mucosal degeneration and ulceration (dotted arrow). Additionally, vacuolated urothelial cells with darkly pigmented nuclei were visible in the urothelium (arrowheads). Inflammatory cells (I) have been abundant in the lamina propria (LP). In addition, dilated congested blood vessels (BV) and widely spread smooth muscle bundles (S) were seen. (**(A, C)** × 100, **(B, D)** × 400)).

**FIGURE 5:**
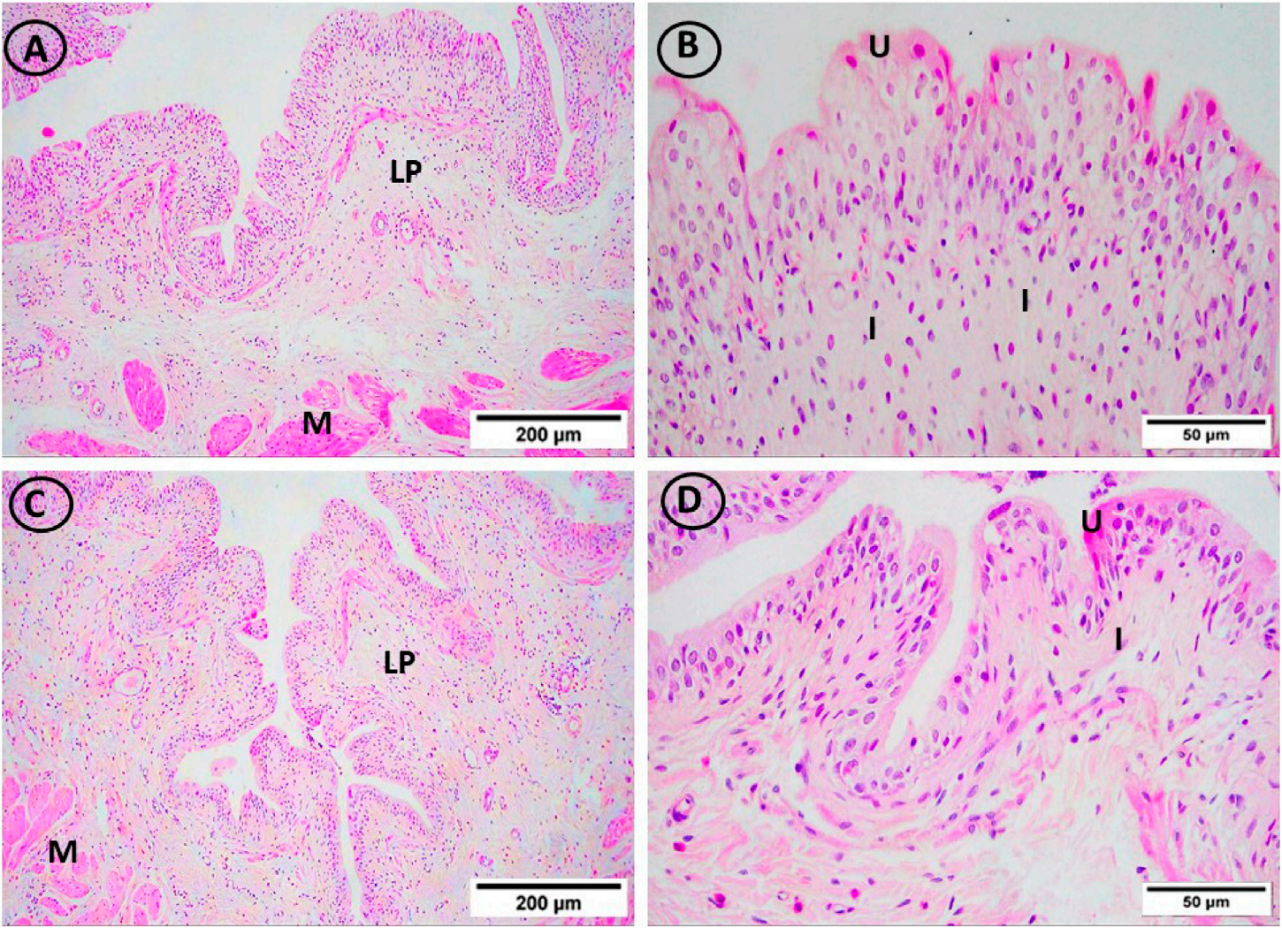
H and E-stained urinary bladder sections. The vitamin D group **(A, B)** and the IF group **(C, D)** both displayed normal lamina propria (LP) and urothelium (U), and smooth muscle bundle organization (M) as well as little infiltrations of inflammatory cells (I). (**(A, C)** × 100, **(B, D)** × 400)).

On lens magnification (x400), rats on HFD developed focal areas of mucosal degeneration and ulceration in bladder sections, and it looked that the transitional epithelium’s thickness had decreased. The nuclei of the vacuolated urothelial cells were highly pigmented. In addition, the lamina propria had a significant infiltration of inflammatory cells. Additionally dilated blood vessels and widely separated smooth muscle bundles were seen. IF and vitamin D groups displayed normal urothelium, smooth muscle bundle organization, and lamina propria with less inflammatory cellular infiltrate (Figures 4 and Figure 5).

### 3.5 Results of histopathological scoring of urinary bladder sections in the study groups

Table 2 provides a descriptive analysis of various histopathological changes. The scores of the histopathological changes (= sum of scores in the 5 examined fields) were significantly higher in HFD group (*p* = 0.000) as compared to the control group. Vitamin D and IF groups showed significant decrease in the histopathological score corelated to HFD group (*p* = 0.000 for both). No significant difference was found between vitamin D and IF groups (*p* = 0.6).

**TABLE 2:** Results of histopathological scoring of all study groups.

	Control	HFD	Vitamin D	IF	*p*-Value
**Mean ± SD**	0.6 ± 0.55^a^	4.6 ± 0.55	0.8 ± 0.44^b^	0.4 ± 0.55^c^	≤ 0.001*

^a^ Significance between control and HFD, groups (*p*-value ≤ 0.001).

^b^ Significance between HFD, and vitamin D groups (*p*-value ≤ 0.001).

^c^ Significance between HFD, and IF, groups (*p*-value ≤ 0.001).

Bold values indicate significant *p* value (< 0.050).

### 3.6 Vitamin D *versus* intermittent fasting effects on the mmunohistochemical expression of AQP-1 in the urinary bladder

**FIGURE 6:**
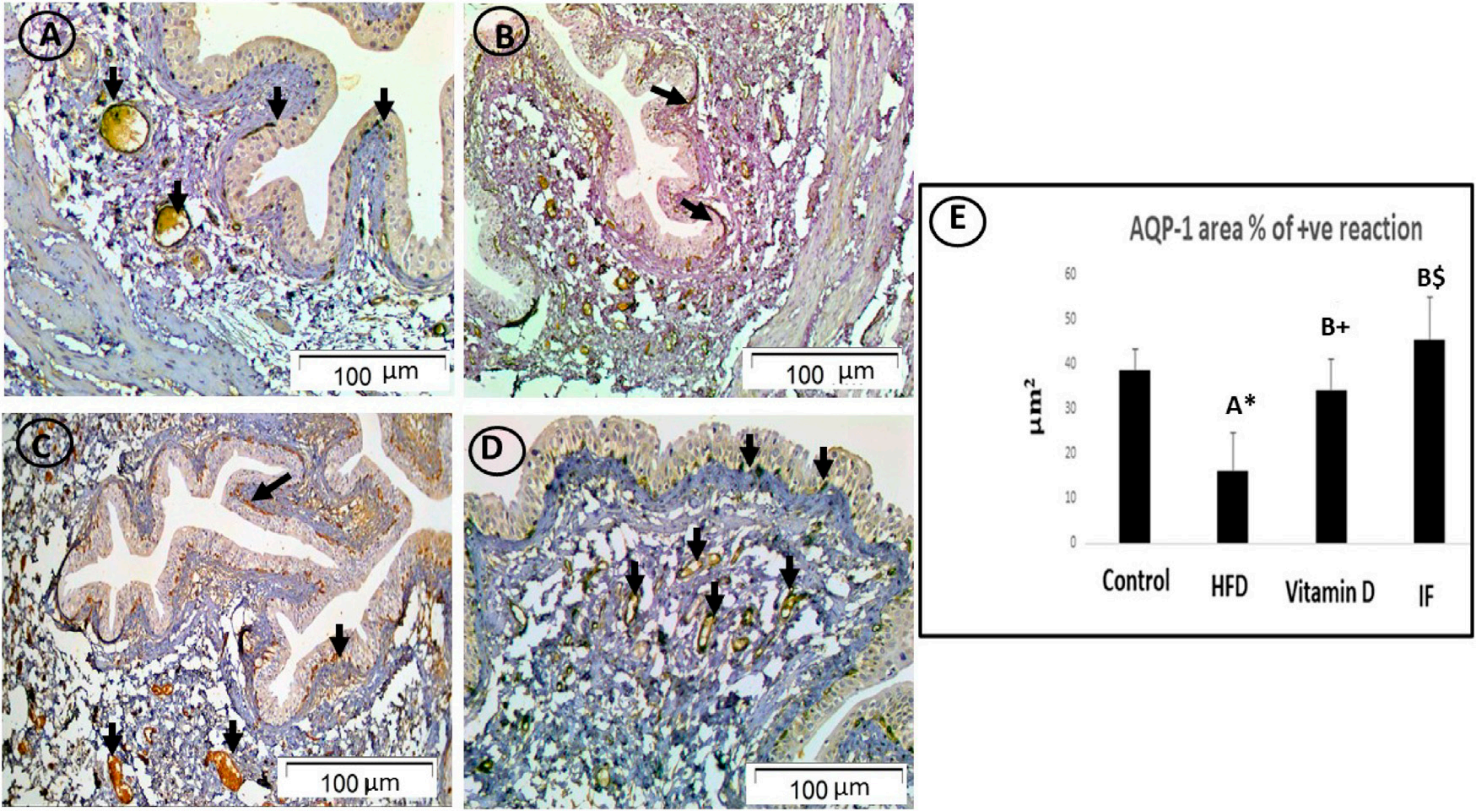
AQP1-immunostained bladder sections. **(A)** In the control group, the cytoplasm of uroepithelium and blood vessel endothelial cells displayed a strong positive immune response (arrows). **(B)** HFD group only had weak immunoreactivity in the uroepithelium (arrow). **(C)** In vitamin D group, endothelial cells of the uroepithelium and blood vessels showed a minimally positive immunological response (arrows). **(D)** The endothelial cells of the uroepithelium and blood vessels in IF group showed a high positive immunological response (arrows), more or less similar to the control group (×200). **(E)** Area % of positive AQP-1 immune reaction. A one-way ANOVA was utilised, followed by a Games Howell post-hoc analysis. A: Relevance for the control group. B: Relevance for the HFD group. +: *p* < 0.05, *: *p* ≤ 0.01, $: *p* ≤ 0.001.

In the control group, the cytoplasm of the uroepithelium and blood vessel endothelial cells showed a highly favourable immune response on lens magnification (x200). While in the HFD group, only the uroepithelium displayed modest immunoreactivity. The uroepithelium and blood vessel endothelial cells, on the other hand, displayed a minimally favourable immunological response in the vitamin D group. Furthermore, endothelial cells and the uroepithelium of rats who underwent IF exhibited a strong favourable immunological response (Figure 6).

### 3.7 Vitamin D *versus* intermittent fasting effects on the expression of AQP-3 urinary bladder tissue

**FIGURE 7:**
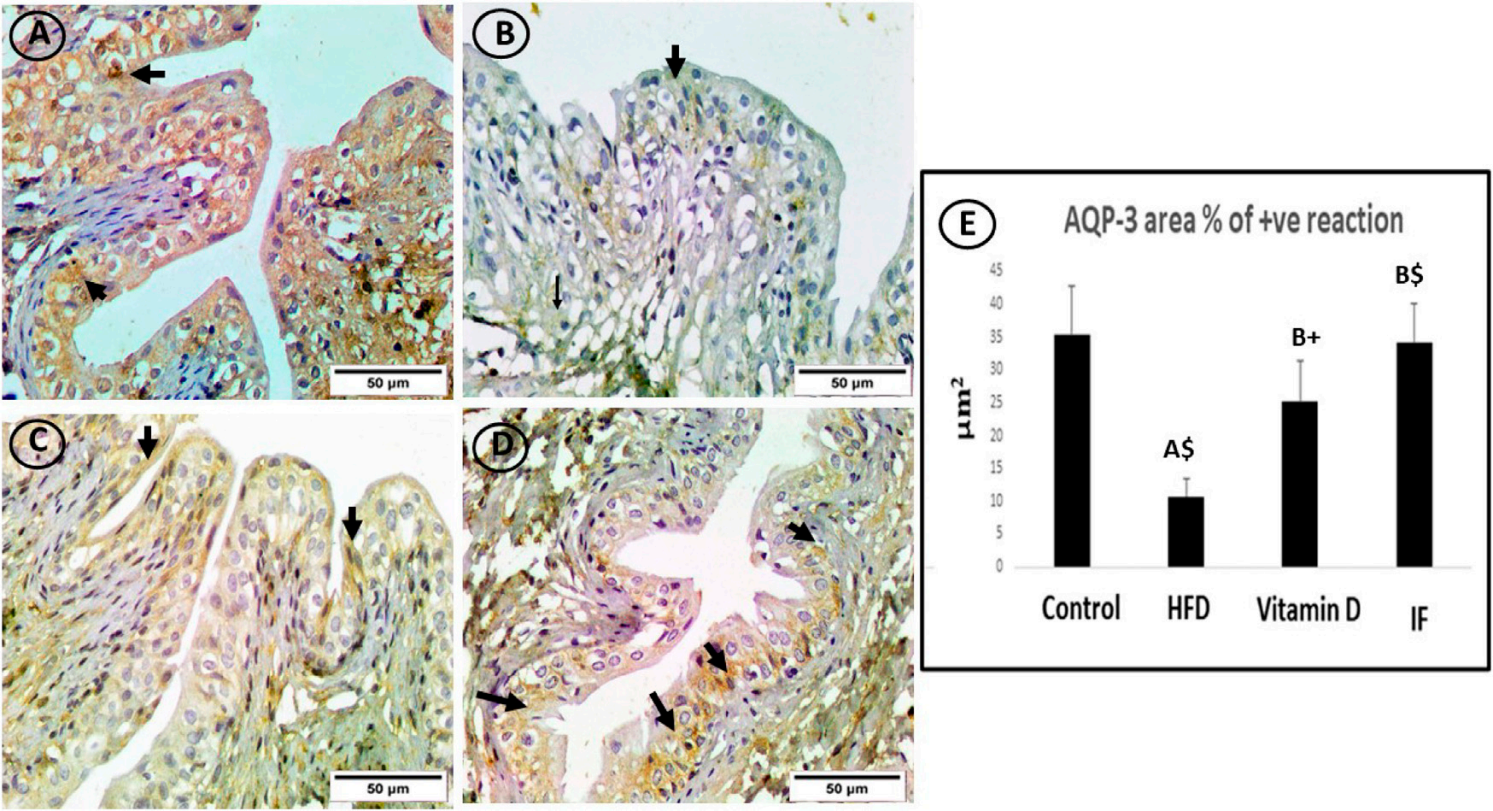
AQP3-immunostained bladder sections. **(A)** The control group showed a strong immune response in the cytoplasm of uroepithelium (arrows). **(B)** Sporadic immunoreactivity was present in the high-fat diet group (arrow). **(C)** The vitamin D group’s uroepithelial cytoplasm responded immunologically in a moderately favourable manner (arrows). **(D)** In the uroepithelium, the IF group showed a significant positive immune response that was comparable to or identical to the control group (arrows) (×400). **(E)** Area % of positive AQP-3 immune reaction. A one-way ANOVA was utilised, followed by a Games Howell post-hoc analysis. A: Relevance for the control group. B: Relevance for the HFD group. +: *p* < 0.05, *: *p* ≤ 0.01, $: *p* ≤ 0.001.

On lens magnification (x400), the cytoplasm of uroepithelium in the control group showed a powerful immune response. On the other hand, the immunoreactivity in the HFD group was negligible. The immunological response was moderately positive in the uroepithelium of the vitamin D group. Surprisingly, the IF group displayed a significantly positive immune response in the uroepithelium (Figure 7).

### 3.8 Vitamin D *versus* intermittent fasting effects on the area percentage of positive AQP-1 and AQP-3 reaction in the urinary bladder

HFD-fed group showed a considerably lower area% of positive AQP-1 and AQP-3 antibodies (*p* = 0.006 and *p* = 0.001, respectively) compared to the control group. The vitamin D group showed a significantly higher area% of positive AQP-1 and AQP-3 immune responses than the HFD group (*p* = 0.02 for both). Additionally, rats on IF had significantly higher area% of positive reactivity for AQP-1 and AQP-3 compared to the HFD group (*p* = 0.001 for both). However, there were no discernible differences between the control group and the groups that received vitamin D or IF (Figure 6E and Figure 7E).

## 4 Discussion

In the current study, rats were given HFD formula for 12 weeks to induce obesity. Clarifying the histological alterations and immunohistochemical expression of AQP-1 and AQP-3 in the urinary bladder of HFD-fed rats was the purpose of the current study. Additionally, comparing the possible benefits of intermittent fasting and vitamin D were investigated.

Numerous metabolic alterations, including impaired lipid metabolism, chronic inflammation, and altered glucose homeostasis, may be made worse by obesity. According to Barretti et al. (2012), obesity is a substantial risk factor for the pathogenesis of the urogenital tract. Furthermore, obesity-induced oxidative stress, which is characterized by an accumulation of reactive oxygen species (ROS), was discovered by Luo et al. (2015) to be a crucial role in the pathophysiology of the urogenital system.

The findings of the current study demonstrated that the HFD group’s serum concentrations of TG, total cholesterol, and LDL cholesterol had drastically increased, indicating dyslipidemia alterations in this group. The control group, however, was significantly fewer.

These results were consistent with those of Woo et al. (2008), who proposed that the dyslipidaemia changes linked to obesity may be caused by an increase in the hepatic triacylglycerol content as a result of an increase in the ingestion of additional non-esterified fatty acids (NEFA).

Vitamin D supplementation showed significantly lower serum levels of TG, LDL, and cholesterol as well as significantly higher HDL levels compared to the HFD group.

These results support those of Yin et al. (2012b), who looked into the effects of vitamin D and found that it significantly reduces the amount of TG produced by HFD by preventing lipogenesis and encouraging lipid oxidation. It also prevents lipid from leaving adipose tissue and eventually accumulating in the liver.

Furthermore, our study found that rats subjected to IF had significantly lower serum levels of TG, LDL, and cholesterol as well as significantly greater serum levels of HDL contrasted with the HFD group. These results corroborated those of Hazzaa et al. (2020), who described these adjustments as being caused by the hypothesis that caloric restriction can improve lipid profile parameters by raising the activity of lipoprotein lipase (LPL), which in turn promotes triglyceride clearance in blood vessels. The activated LPL also speeds up the breakdown of triglyceride-rich lipoproteins, transferring esters, apoproteins, and phospholipids to produce HDL (Chen et al., 2015). Apolipoprotein A-1 may also be at its highest during intermittent fasting, which lowers calorie intake and raises HDL concentrations (Das and Mishra, 2012).

Moving on to our findings regarding the evaluation of oxidative stress indicators, the HFD group had a significantly higher level of MDA and NO and a significantly lower level of GSH in comparison to the control group.

Our data supported the conclusion of Emami et al. (2016) that HFD-induced obesity encourages the generation of ROS. Nitric oxide, a well-known vasoactive compound, has also been related to the emergence of diseases including diabetes and obesity, according to Cheng and Harris (2014). MDA is an oxidative cell factor that results from lipid peroxidation in the cell membrane, although GSH is essential for preventing oxidative damage to tissues (Noeman et al., 2011).

Additionally, obesity may cause cumulative, slow-onset cell damage that heightens lipid peroxidation. Particularly, damaged cells release tumor necrosis factor alpha (TNF-a), which generates ROS and causes lipid peroxidation. Lipid peroxidation may increase when free fatty acid bioavailability increases. Additionally, altered oxidant-antioxidant balance could be caused by obesity-related hypertriglyceridemia (Farshad et al., 2007).

According to the current study, when compared to HFD group, vitamin D group had significantly lower levels of MDA and NO and significantly higher levels of GSH.

Al-Daghri et al. (2018) further stated that ROS elimination pathway and intracellular pool of reduced GSH were increased, and vitamin D enhanced glutathione reductase. Also, vitamin D administration could block the messenger RNA of inducible nitric oxide synthase (iNOS) and that NO generation was also decreased.

The present study showed that IF significantly decreased MDA and NO levels and increased GSH level compared to HFD group.

This outcome was consistent with the findings of Lee et al. (2006), who claimed that caloric restriction significantly decreased the production of ROS and the consumption of cellular oxygen in the mitochondria of rat muscle. Additionally, fasting significantly reduced the amount of oxidative DNA damage (Hazzaa et al., 2020). Furthermore, it was noted by Gnoni et al. (2021) that IF decreased the amount of messenger ribonucleic acid (mRNA) that encodes iNOS in the rat hippocampus. Additionally, Khouchab et al. (2020) discovered that prolonged IF enhances both nonendothelial and endothelial microvascular activities.

As indicated by Li et al. (2013), who discovered that a 40% reduction in caloric intake lowers oxidative stress in several parts of the brain, IF-induced caloric restriction also enhances the level of the brain’s redox state. They also mentioned that IF has a neuroprotective impact and boosts the hippocampus’ resilience to excitotoxic stress, which may be because of mitochondrial reprogramming that lowers oxidant production.

The current study examined urine bladder specimens and revealed some histopathological deteriorations in the HFD group. There were specific areas of mucosal degeneration and ulceration, a thinned urothelium, and some uroepithelial cells that have vacuoles with diffuse inflammatory cellular infiltration.

These findings were in line with research by Elmas et al. (2022), which shown that ectopic lipid buildup increases systemic inflammation by triggering the release of pro-inflammatory mediators and the recruitment of macrophages.

Oberbach et al.'s (2014) explanation for the degenerative alterations also included the buildup of ROS, which alter protein function through carbonylation. Degeneration of the urinary bladder was caused by these carbonylated proteins.

In addition, Spoto and Zoccali (2013) reported that there was a connection between obesity and a chronic pro-inflammatory condition. The increase in macrophage infiltration and/or the increased expression of genes linked to adipose tissue inflammation were blamed for this inflammation.

The current study declared that vitamin D administration and IF restored the architecture of the lamina propria, smooth muscle bundles, and urothelium. The inflammatory response was also minimally there.

These results were consistent with those made by Huang (2016), who found that intestinal cells, keratinocytes, and urine bladder have all been shown to produce antimicrobial peptides in response to vitamin D administration. Furthermore, TNF and pro-inflammatory cytokines like IL-6 as well as lipid peroxidation products were shown to be reduced by vitamin D, which is thought to have anti-inflammatory properties, according to Kheder et al. (2017). Furthermore, according to Almerighi et al. (2009), vitamin D’s primary immunomodulatory and anti-inflammatory effects come from its direct impact on T cells, which inhibits the synthesis of pro-inflammatory cytokines and promotes the expression of anti-inflammatory cytokines. Intermittent fasting causes mitochondrial reprogramming that lowers the generation of oxidants (Li et al., 2013).

Furthermore, the nuclear factor kappa-B signalling pathway, which is a crucial regulator of downstream variables such as TNF- and IL-6, is inhibited by IF (Liu et al., 2019). This is in line with the findings of Zouhal et al. (2020), who claimed that IF could reduce CRP and TNF- while largely eradicating inflammation triggered by adipose tissue.

The current study evaluated the expression of AQP-1 and AQP-3 in urinary bladder samples. Compared to the control group, the HFD group showed a very little immunoreactivity of AQP-1 and AQP-3 in the uroepithelium.

This finding was in line with that made by Gregoire et al. (2015), who evaluated AQP in the livers of rats fed an HFD and found that AMP-activated protein kinase (AMPK), a protein essential in maintaining the equilibrium of lipid and carbohydrate metabolism, serves as an energy sensor. The mRNA levels of AQP were shown to be significantly reduced in primary culture of murine hepatocytes by AMPK activator.

Additionally, mice given HFD showed a considerable downregulation of the AQP-4 gene in the kidney, which Kuhns and Pluznick (2018) hypothesized that it was caused by an increase in cellular ROS generation resulting from lipid metabolic disorders, similarly to AQP-3 expression. Furthermore, hypercholesterolemia induced tubular inflammation, necroptosis, and decreased AQP expression in the kidney (Kong et al., 2020).

In contrast to the HFD group, the current study demonstrated that the vitamin D group exhibited a mildly positive immune response for both AQP-1 and AQP-3 in the uroepithelium and the endothelial cells of blood vessels.

Our findings were supported by Fu et al. (2019), who found that vitamin D increases the expression of AQP--1 in mouse kidneys. Furthermore, Park et al. (2010) discovered that the vitamin D analogue boosted the expression of AQP-1 in rats with renal damage triggered by gentamicin.

The current study revealed that, in contrast to the HFD group, the IF group had a robust positive immunological response to both AQP-1 and AQP-3 in the uroepithelium and endothelial cells.

This result was consistent with the findings of Neumann et al. (2016), who found that endogenous hormones are in control of AQP-1 stimulation by fasting. The significance of glucocorticoid receptors (GREs) in regulating the expression of water channels was further supported by this discovery.

The present study highlighted that IF offered better results than vitamin D in HFD-induced obesity in terms of biochemical, histopathological, molecular, and immunohistochemical parameters.

Our declaration was in line with Longo and Mattson (2014), who concluded that IF can enhance cellular resistance to diseases using different signalling pathways. Furthermore, Sharsher et al. (2022) discovered that IF causes weight loss and fat loss by lowering ROS production, raising cellular stress, and lowering inflammation.

## 5 Conclusion

The results of the current study show that IF and vitamin D can both reverse bladder degeneration induced by HFD. Compared to vitamin D, IF had a stronger protective effect on biochemical, histological, immunohistochemical, and molecular parameters.

## 6 Limitations of the study

As a result, the current study demonstrated that intermittent fasting, as well as vitamin D, are considered candidates to be used in the obesity regimen. However, to support the findings of our experiment, clinical trials are suggested. Additionally, further study is required to properly comprehend how vitamin D and intermittent fasting influence AQPs expression under several experimental settings.

## Data Availability

The raw data supporting the conclusion of this article will be made available by the authors, without undue reservation.
